# Exploration of Optimal Synergistic Treatment Strategies of Postoperative Radiotherapy and Immunotherapy in Early-Stage Breast Cancer

**DOI:** 10.3390/cancers18071145

**Published:** 2026-04-02

**Authors:** Qingyao Shang, Hanyu Wang, Yan Zhuang, Jennifer K. Plichta, Samantha M. Thomas, Meishuo Ouyang, Sheng Luo, Xin Wang

**Affiliations:** 1 Department of Breast Surgical Oncology, National Cancer Center/National Clinical Research Center for Cancer/Cancer Hospital, Chinese Academy of Medical Sciences and Peking Union Medical College, Beijing 100021, China; 2 Department of Biostatistics & Bioinformatics, Duke University School of Medicine, Durham, NC 27705, USA; 3 Duke Cancer Institute, Duke University School of Medicine, Durham, NC 27710, USA; 4 Department of Surgery, Duke University School of Medicine, Durham, NC 27707, USA

**Keywords:** breast cancer, radiotherapy, immunotherapy, treatment sequencing, abscopal effect

## Abstract

**Background**: The optimal sequencing of radiotherapy and immunotherapy in early-stage breast cancer remains uncertain. Although synergistic interactions between radiotherapy and immunotherapy have been widely reported, most available evidence derives from advanced disease with high tumor burden. Whether treatment sequencing influences outcomes in postoperative adjuvant therapy has not been well defined. **Methods**: Patients with stage I–III human epidermal growth factor receptor 2 (HER2)-negative breast cancer who underwent surgery followed by both adjuvant radiotherapy and immunotherapy were identified from the National Cancer Database. According to treatment initiation dates, patients were classified into immunotherapy-first and radiotherapy-first groups. Overall survival was compared using Kaplan–Meier analysis and weighted Cox regression. Baseline imbalances were adjusted using inverse probability of treatment weighting. Prespecified subgroup analyses were conducted based on adjuvant chemotherapy status and radiotherapy fractionation regimen. A sensitivity analysis was performed in an independent cohort of stage IV inoperable patients. **Results**: A total of 3813 patients were included. Immunotherapy-first sequencing was associated with improved overall survival compared with radiotherapy-first sequencing after weighted (HR 0.71; 95% CI 0.56–0.89). The survival benefit was most evident among patients receiving adjuvant chemotherapy (HR 0.63; 95% CI 0.48–0.84), whereas no significant difference was observed in patients without chemotherapy (HR 1.01; 95% CI 0.71–1.44). Subgroup analysis according to radiotherapy fractionation demonstrated a significant advantage of immunotherapy-first sequencing in patients treated with conventional fractionation radiotherapy (HR 0.58; 95% CI 0.36–0.92), but not in those receiving hypofractionated radiotherapy (HR 0.44; 95% CI 0.17–1.13). In stage IV inoperable patients, no survival difference was detected between sequencing strategies (HR 1.04; 95% CI 0.88–1.23). **Conclusions**: For postoperative patients with low residual tumor burden, particularly those receiving adjuvant chemotherapy, immunotherapy-first strategy may provide stronger synergistic effects and lead to improved survival. In addition, conventional fractionation radiotherapy with lower doses per fraction appears to facilitate more effective interaction with immunotherapy compared with hypofractionated regimens. Prospective trials are needed for further validation.

## Simple Summary

Radiotherapy and immunotherapy have demonstrated synergistic antitumor effects, yet the optimal strategy for integrating these two modalities in patients with early-stage breast cancer remains unclear. In this study, we analyzed real-world data from a large national database to compare the sequencing of immunotherapy and radiotherapy after surgery. Our results showed that starting immunotherapy first was associated with better overall survival, particularly among patients receiving adjuvant chemotherapy and those treated with conventional fractionated radiotherapy. These findings suggest that, for early-stage breast cancer with low residual tumor burden, an immunotherapy-first strategy may provide greater clinical benefit and offer practical guidance for optimizing postoperative treatment planning.

## 1. Introduction

Breast cancer remains a leading cause of cancer-related mortality among women worldwide [1]. Treatment strategies are determined by tumor stage and biological characteristics, incorporating multimodal approaches such as surgery, chemotherapy, endocrine therapy, targeted therapy, radiotherapy, and immunotherapy [2]. Among these, radiotherapy is a well-established modality for reducing locoregional recurrence, particularly in patients with high-risk features such as positive lymph nodes or large tumor size [3]. Immunotherapy, especially immune checkpoint inhibitors, has demonstrated promising efficacy in specific subgroups of breast cancer, most notably in HER2-negative disease, including triple-negative breast cancer and tumors with high tumor mutational burden [4,5].

Given the increasing use of immunotherapy in HER2-negative breast cancer, its integration with other treatment modalities has become an important clinical consideration. Current clinical practice and guidelines support the use of radiotherapy in combination with systemic therapies, including immunotherapy, in appropriate patient populations. The combination of radiotherapy and immunotherapy has been proposed as a strategy to enhance anti-tumor efficacy, as radiotherapy not only induces direct cytotoxic effects but also promotes systemic immune activation by enhancing antigen presentation and modulating the tumor microenvironment [6,7]. This provides a strong biological rationale for potential synergy between these two modalities.

However, most existing evidence regarding the synergy between radiotherapy and immunotherapy is derived from metastatic settings, where high tumor burden may amplify immune responses [8]. In contrast, in postoperative early-stage breast cancer, where residual tumor burden is relatively low and treatment intent is curative, the optimal integration of radiotherapy and immunotherapy remains unclear. In particular, while the combination of radiotherapy and immunotherapy has been increasingly incorporated into clinical practice, the optimal integration of these modalities, including their sequencing and radiation dosing, remains unclear. These factors may critically influence therapeutic efficacy. Therefore, this study aims to evaluate the optimal strategy for combining radiotherapy and immunotherapy in patients with early-stage HER2-negative breast cancer.

## 2. Materials and Methods

This retrospective cohort study was conducted using data from the National Cancer Database (NCDB, 2022 version) [9]. Adult patients with stage I–III HER2-negative breast cancer who underwent definitive surgery followed by both adjuvant radiotherapy and immunotherapy were eligible for inclusion.

Patients were included if they met all of the following criteria: (1) histologically confirmed invasive breast carcinoma; (2) pathologic stage I–III disease at diagnosis; (3) HER2-negative status; (4) receipt of both postoperative radiotherapy and immunotherapy within 12 months after surgery; and (5) complete records of treatment initiation dates and survival outcomes. Exclusion criteria were limited to: (1) evidence of distant metastasis at diagnosis (M1 disease); (2) missing or indeterminate initiation dates for radiotherapy or immunotherapy; and (3) incomplete follow-up or survival information.

Treatment sequencing was determined based on the recorded start dates of radiotherapy and immunotherapy in the NCDB. The index date was defined as the initiation date of the first modality received after surgery. Immunotherapy was identified using variables within the “Systemic Therapy” field of the NCDB. Because the NCDB does not capture detailed drug-level information, specific immune checkpoint inhibitors (e.g., atezolizumab or durvalumab) could not be distinguished. Radiotherapy was identified using variables within the “Radiation” field of the NCDB. Patients were classified into two groups: the radiotherapy-first group (RI), defined as initiation of radiotherapy prior to immunotherapy, and the immunotherapy-first group (IR), defined as initiation of immunotherapy prior to radiotherapy. Because only an extremely small proportion of patients (0.5%) initiated radiotherapy and immunotherapy on the same day, these cases were excluded to avoid ambiguity in sequencing classification.

Radiotherapy fractionation was categorized based on dose per fraction and total dose recorded in the NCDB. Based on definitions reported in previous randomized controlled trials and clinical guidelines [10,11], we adopted a relatively broad classification to reflect the biological characteristics of different radiotherapy fractionation patterns. Conventional fractionation was defined as single dose ≤ 2 Gy with a total dose ≥ 50 Gy, representing the low-dose-per-fraction regimen. Hypofractionated fractionation was defined as single dose > 2 Gy with a total dose < 45 Gy, representing higher dose-per-fraction schedules. The primary outcome was overall survival (OS), defined as the time from the index date to death from any cause. Patients who were alive at last follow-up were censored at that time.

To further explore whether the effect of sequencing differed according to disease burden, a prespecified sensitivity analysis was performed using an independent cohort of patients with newly diagnosed stage IV HER2-negative breast cancer. These patients were required to have received both radiotherapy and immunotherapy without prior surgical resection. The definitions of sequencing (RI vs. IR) and all analytic methods were identical to those used in the primary cohort.

### Statistical Analysis

Baseline characteristics of patients in the RI and IR groups were summarized and compared using standardized mean differences (SMD), with an absolute SMD > 0.10 considered indicative of clinically meaningful imbalance.

To adjust for potential confounding, inverse probability of treatment weighting (IPTW) was used as the primary analytic approach, and propensity score matching (PSM) was performed as a secondary method for comparison [12]. Propensity scores representing the probability of receiving IR sequencing were estimated using multivariable logistic regression incorporating the covariates listed in Table 1, including: age at diagnosis, race, insurance status, area-level median income, histologic subtype, hormone receptor status, pathologic stage, tumor grade, Charlson–Deyo comorbidity score, type of surgery, receipt of adjuvant chemotherapy, receipt of neoadjuvant therapy. Stabilized IPTW weights were calculated to improve estimation stability. Extreme weights were truncated at the 1st and 99th percentiles. After weighting, covariate balance was reassessed using SMDs and graphical diagnostics.

PSM was conducted using 1:1 nearest-neighbor matching without replacement and a caliper width of 0.2 of the standard deviation of the logit of the propensity score. PSM results were used only for descriptive comparison and validation of robustness.

OS was estimated using Kaplan–Meier methods, and differences between sequencing groups were compared using the log-rank test. Weighted Cox proportional hazards regression models were used to estimate hazard ratios (HRs) and 95% confidence intervals (CIs). The proportional hazards assumption was evaluated using Schoenfeld residuals.

Subgroup analyses were first performed across a broad range of clinical variables using a prespecified forest plot. Based on these results, detailed subgroup analyses were subsequently conducted for two clinically important factors: receipt of adjuvant chemotherapy and radiotherapy fractionation regimen. Interaction terms between treatment sequence and subgroup variables were incorporated into Cox models to formally test for effect modification.

Patients with missing values for key variables were excluded from the relevant analyses. All statistical analyses were performed using R software (version 4.4.1; R Foundation for Statistical Computing, Vienna, Austria). Two-sided *p* values < 0.05 were considered statistically significant.

## 3. Results

A total of 3813 patients with stage I–III HER2-negative breast cancer who received both adjuvant radiotherapy and immunotherapy after surgery were identified from the NCDB database (Table 1). Among them, 923 patients (24.2%) were classified into the RI group and 2890 patients (75.8%) into the IR group according to the sequence of treatment initiation.

Before adjustment, substantial baseline differences were observed between the two groups. Patients in the RI group were older than those in the IR group (mean age 59.52 vs. 56.90 years; SMD = 0.22). A higher proportion of stage III disease was noted in the IR group (21.7% vs. 16.0%; SMD = 0.28), indicating a relatively higher baseline risk profile. In addition, the RI group was less likely to receive adjuvant chemotherapy compared with the IR group (37.9% vs. 88.4%; SMD = 1.23). Imbalances were also observed in nodal status, tumor grade, and insurance type, suggesting potential selection bias in treatment sequencing.

To minimize these baseline differences, both IPTW and PSM approaches were implemented. After adjustment, covariate balance improved markedly in both methods. IPTW achieved superior performance, with all post-weighting SMDs reduced to <0.10 (Figures S1 and S2). Given the more robust balance achieved with IPTW, all subsequent outcome analyses were performed using the IPTW-adjusted cohort. The detailed distribution of baseline characteristics before and after adjustment is presented in Table 1.

**Figure 1:**
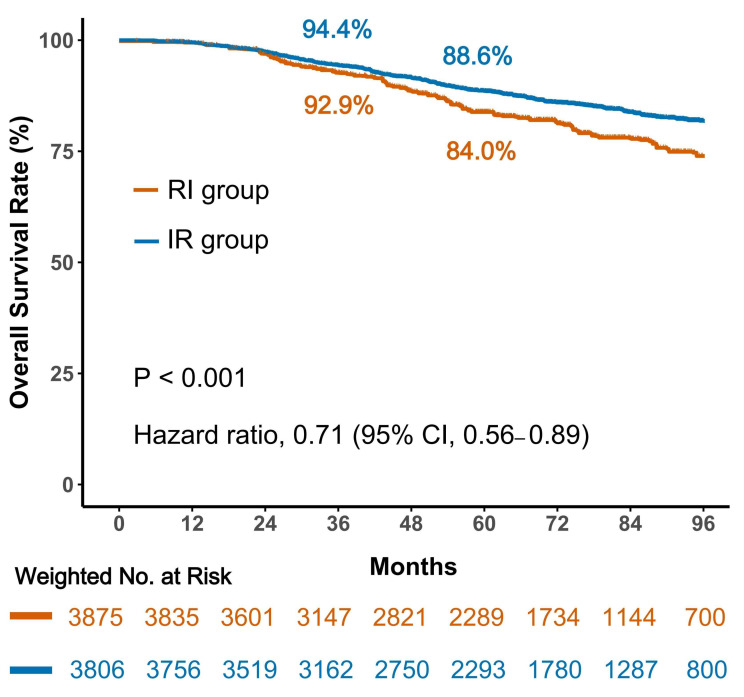
Overall survival according to treatment sequencing. Kaplan–Meier curves comparing overall survival between patients treated with immunotherapy-first sequencing (IR group) and those treated with radiotherapy-first sequencing (RI group) after adjustment.

In the IPTW-adjusted survival analysis, a significant association between treatment sequencing and OS was observed. Kaplan–Meier estimates demonstrated that patients in the IR group had a significant survival advantage compared with those in the RI group (HR = 0.71; 95% CI, 0.56–0.89; *p* < 0.001; Figure 1). Correspondingly, 5-year OS rates were 88.6% in the IR group and 84.0% in the RI group, indicating a clinically meaningful absolute survival difference.

**Figure 2:**
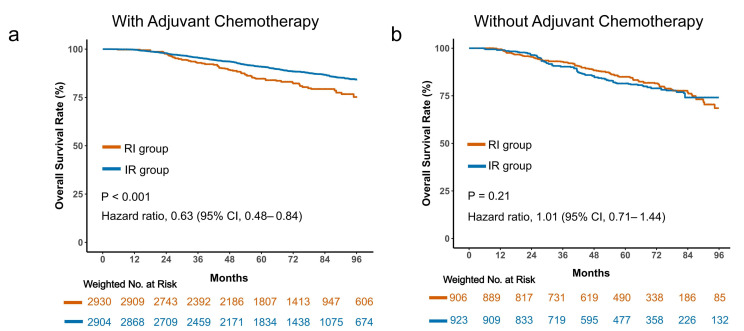
Subgroup analysis based on receipt of adjuvant chemotherapy. Kaplan–Meier survival curves comparing sequencing strategies stratified by adjuvant chemotherapy status. Survival outcomes are presented separately for patients who received adjuvant chemotherapy (**a**) and those who did not (**b**).

To explore whether the observed sequencing effect was consistent across patient subgroups, prespecified subgroup analyses were performed (Figure S3). The survival benefit associated with the IR sequence remained directionally consistent across most clinical variables, including age groups, nodal status, and pathologic stage. Importantly, a significant interaction was observed according to receipt of adjuvant chemotherapy. Among patients treated with chemotherapy, the IR sequence was associated with a pronounced reduction in mortality risk (HR = 0.63; 95% CI, 0.48–0.84; *p* < 0.001; Figure 2a). In contrast, no significant difference was detected in patients who did not receive chemotherapy (HR = 1.01; 95% CI, 0.71–1.44; *p* = 0.21; Figure 2b). These results suggest that tumor burden and intensity of systemic therapy may play important roles in modifying the benefit of sequencing strategies.

**Figure 3:**
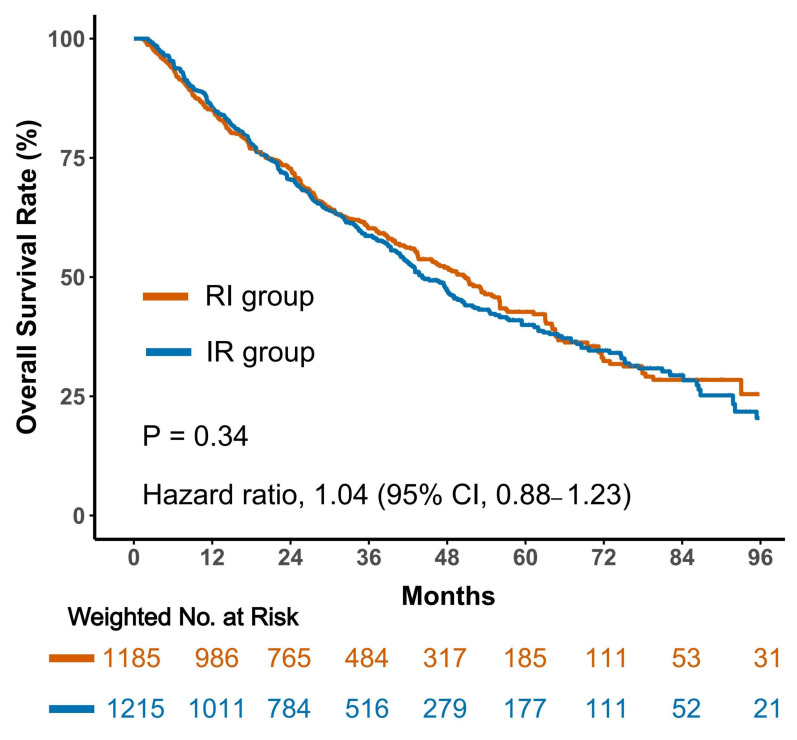
Sensitivity analysis in stage IV inoperable patients. Kaplan–Meier curves comparing overall survival between sequencing strategies in an independent cohort of patients with newly diagnosed stage IV inoperable HER2-negative breast cancer treated with both radiotherapy and immunotherapy.

To further evaluate the potential impact of tumor burden, a sensitivity analysis was conducted in an independent cohort of stage IV inoperable patients who received both radiotherapy and immunotherapy. In this high-burden population, no significant difference in OS was observed between the two sequencing approaches (HR = 1.04; 95% CI, 0.88–1.23; *p* = 0.34; Figure 3). Additional analyses stratified by metastatic sites, including brain, visceral, and bone metastases, similarly failed to demonstrate statistically significant sequencing effects. Nevertheless, a trend favoring the RI sequence was noted in several subgroups with extensive disease burden (Figure S4), supporting the hypothesis that optimal sequencing may vary according to tumor burden.

**Figure 4:**
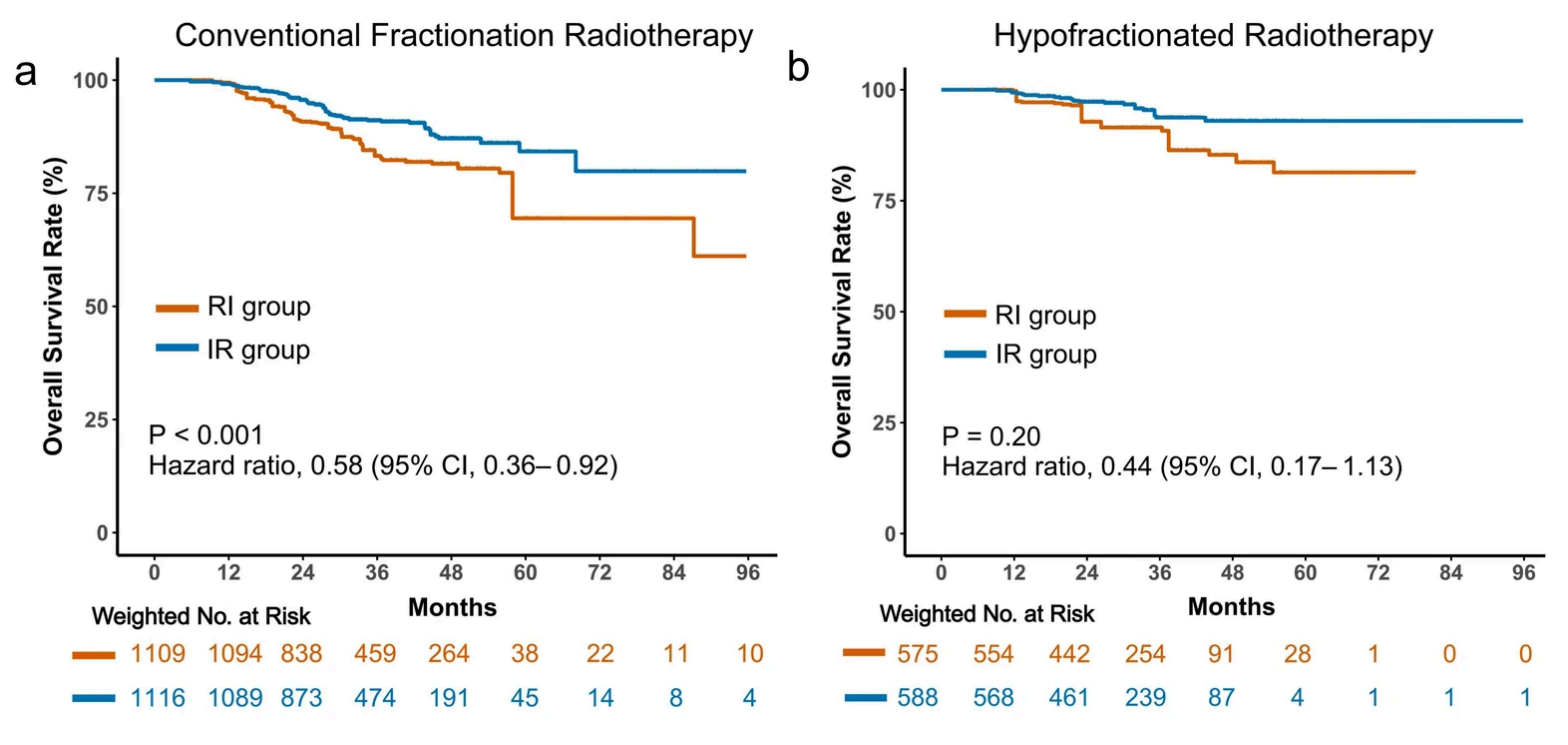
Subgroup analysis by radiotherapy fractionation regimen. Kaplan–Meier curves comparing treatment sequencing among patients receiving conventional fractionation radiotherapy (**a**) and those receiving hypofractionated radiotherapy (**b**).

We next investigated whether the radiotherapy fractionation regimen modified the association between sequencing and survival among patients treated with the RI sequence. In this subgroup, patients who received conventional fractionation radiotherapy experienced a significant survival benefit (HR = 0.58; 95% CI, 0.36–0.92; *p* < 0.001; Figure 4a). In contrast, among those treated with hypofractionated radiotherapy, no significant survival difference was observed (HR = 0.44; 95% CI, 0.17–1.13; *p* = 0.20; Figure 4b). These findings suggest that the interaction between radiotherapy and immunotherapy may be more pronounced when radiotherapy is delivered using a conventional fractionation schedule, potentially due to differences in immune activation dynamics.

## 4. Discussion

In this large real-world cohort of patients with early-stage HER2-negative breast cancer, treatment sequencing between radiotherapy and immunotherapy was found to be significantly associated with OS. Specifically, an immunotherapy-first strategy was associated with superior survival compared with radiotherapy-first approach. This advantage was particularly evident among patients receiving adjuvant chemotherapy and those treated with conventional fractionation radiotherapy, whereas no sequencing effect was observed in the stage IV inoperable patients. These findings suggest that the optimal integration of radiotherapy and immunotherapy is highly dependent on tumor burden.

Extensive preclinical and clinical studies have elucidated multiple biological mechanisms through which radiotherapy can potentiate the efficacy of immunotherapy. Radiotherapy has been shown to induce immunogenic cell death, promote the release of tumor-associated antigens, upregulate MHC class I expression, and activate the cGAS-STING pathway, thereby enhancing type I interferon signaling and dendritic-cell-mediated T-cell priming [7]. Moreover, radiotherapy can increase infiltration of cytotoxic T lymphocytes into the tumor microenvironment and upregulate PD-L1 expression, providing a strong biological rationale for combining radiotherapy with immune checkpoint blockade [13]. These mechanistic insights have been translated into meaningful clinical benefit, most notably in the PACIFIC trial, which enrolled patients with stage III unresectable non-small cell lung cancer who had completed chemoradiotherapy [14]. Consolidation durvalumab significantly improved both progression-free and overall survival compared with placebo. Importantly, most of the mechanistic and clinical evidence supporting the synergy between radiotherapy and immunotherapy has been derived from studies of locally advanced or metastatic unresectable tumors characterized by substantial residual disease burden. This pattern is consistent with the findings of the sensitivity analysis in the present study, which focused on patients with stage IV inoperable breast cancer. In this high tumor burden population, radiotherapy followed by immunotherapy showed a numerically more favorable survival trend, although the difference did not reach statistical significance.

In contrast, early-stage postoperative breast cancer represents a fundamentally different clinical condition [15]. After surgery and systemic therapy, residual tumor burden is typically minimal, restricting the extent of radiotherapy-induced tumor cell death and thereby limiting the generation and release of tumor neoantigens [16]. Under such circumstances, the radiotherapy-driven mechanisms of immune activation described in metastatic disease may be less operative. The primary analysis in the present study demonstrates that, in early-stage disease, an immunotherapy-first strategy confers a clear survival benefit. This observation suggests that initiating immunotherapy prior to radiotherapy may establish a preactivated systemic immune environment, enabling subsequent radiotherapy to more effectively amplify antitumor immunity rather than serving as the primary immune-initiating modality. Preclinical studies have demonstrated that radiotherapy can induce systemic antitumor immune responses capable of controlling distant lesions, particularly when combined with immune checkpoint inhibitors, a phenomenon referred to as the abscopal effect [17,18]. Increasing evidence indicates that this immune-mediated regression of tumors outside the irradiated field represents a central mechanism underlying the synergistic interaction between radiotherapy and immunotherapy [19,20]. Delivering immunotherapy first may activate and prime the immune microenvironment, thereby facilitating a more robust abscopal response following radiotherapy.

In addition, the present study found that the survival advantage of immunotherapy-first sequencing was more pronounced among patients receiving adjuvant chemotherapy. Adjuvant chemotherapy can further reduce residual disease after surgery, resulting in a lower overall tumor burden [21]. Thus, administering immunotherapy after chemotherapy may take advantage of chemotherapy-induced immune activation, as chemotherapy has been shown to increase tumor-infiltrating lymphocytes and to enhance antitumor immune responses, creating a more favorable immunologic microenvironment for subsequent radiotherapy to elicit systemic immune effects [22]. Chemotherapy may also exert immunomodulatory effects, including depletion of immunosuppressive regulatory T cells and myeloid-derived suppressor cells, as well as enhancement of antigen presentation [23,24]. These mechanisms could further potentiate the synergistic interaction between immunotherapy and radiotherapy.

Radiotherapy dose and fractionation have been shown to play a critical role in shaping the immune consequences of radiotherapy [25]. Multiple studies have shown that conventional or moderately fractionated radiotherapy more effectively stimulates antitumor immune responses than single high-dose irradiation and provides greater potential for synergistic interaction with immune checkpoint inhibitors [26,27]. Fractionated low-dose radiotherapy has been reported to facilitate dendritic-cell activation, increase T-cell infiltration, and promote a more favorable immune microenvironment. In contrast, very high doses per fraction can activate immunosuppressive pathways, such as TREX1-mediated degradation of cytosolic DNA, which attenuates cGAS-STING signaling and limits radiation-induced immune activation [28,29,30]. Immunologic effects of radiotherapy are known to be dose dependent, and different fractionation schedules may influence the interaction with immunotherapy. In the present study, a survival benefit of immunotherapy-first sequencing was observed predominantly among patients treated with conventional fractionation radiotherapy, whereas no statistically significant difference was observed in the hypofractionated subgroup. However, given the relatively small sample size in the hypofractionated subgroup, particularly in the RT-first group, this finding should be interpreted with caution and may reflect limited statistical power rather than a true biological difference. Taken together with prior evidence, these results may suggest a potential role of fractionation in modulating the interaction between radiotherapy and immunotherapy, although further validation is warranted. Taken together, our results suggest a tumor burden-dependent paradigm for sequencing radiotherapy and immunotherapy in breast cancer. In high-burden metastatic disease, initial radiotherapy may be necessary to generate sufficient antigen release, whereas in early-stage postoperative patients, priming the immune system with immunotherapy before radiotherapy may better harness systemic antitumor immunity and enhance abscopal-like effects.

The substantial imbalance in adjuvant chemotherapy use between the two groups warrants careful interpretation. Patients in the immunotherapy-first group were more likely to receive chemotherapy, which is consistent with current clinical practice, where immune checkpoint inhibitors are typically administered in combination with chemotherapy in HER2-negative breast cancer, particularly in triple-negative disease. In contrast, patients receiving radiotherapy first may represent a different treatment pathway, potentially reflecting differences in treatment priorities or overall risk assessment. Although IPTW was applied to balance measured covariates, residual confounding cannot be completely excluded. Therefore, the observed associations should be interpreted with caution and considered hypothesis-generating.

Several limitations of this study should be acknowledged. First, the retrospective design introduces the possibility of residual confounding despite the use of IPTW adjustment. Although extensive baseline variables were incorporated into the propensity score model, unmeasured factors related to treatment selection may still exist. Second, the NCDB lacks information on important immunological biomarkers, such as PD-L1 expression and tumor-infiltrating lymphocytes, which limits mechanistic stratification of immunotherapy response. In addition, the database does not provide information on the specific immune checkpoint inhibitors or treatment regimens used, preventing evaluation of potential differences between individual agents. Third, detailed treatment-related variables, including toxicity profiles, recurrence patterns, and disease-free survival outcomes, are not available in the NCDB dataset, which precludes assessment of additional clinically relevant endpoints. Fourth, the stage IV cohort used in the sensitivity analysis represents a population with substantially higher tumor burden but differs fundamentally from postoperative early-stage breast cancer in terms of disease biology, treatment intent, and clinical management. Therefore, this analysis should be interpreted as an exploratory reference rather than a direct surrogate for high tumor burden in early-stage disease. Finally, although treatment sequencing was determined based on recorded initiation dates, potential biases inherent to observational database studies cannot be completely excluded. Nevertheless, the large sample size and consistent findings across multiple analytical approaches provide meaningful real-world evidence regarding the potential interaction between radiotherapy and immunotherapy sequencing in early-stage breast cancer.

## 5. Conclusions

Our study indicates that in early-stage HER2-negative breast cancer, an immunotherapy-first sequencing strategy is associated with improved survival, particularly among patients with lower tumor burden and those treated with conventional fractionation radiotherapy. These findings highlight the importance of considering tumor burden, immune context, and radiation delivery parameters when integrating radiotherapy and immunotherapy. Prospective trials incorporating immune biomarkers are needed to validate these observations and to define personalized sequencing strategies.

## Figures and Tables

**Table 1:** Baseline Characteristic.

Characteristic	RI Group	IR Group	SMD	RI Group	IR Group	SMD
	Unweighted	Unweighted		IPTW	IPTW	
Number	923	2890		3874.92	3805.94	
Age (mean (SD))	59.52 (12.23)	56.90 (11.88)	0.22	57.14 (11.92)	57.44 (12.10)	0.03
Race (%)			0.06			
Asian	35 (3.8)	104 (3.6)		155.3 (4.0)	138.4 (3.6)	0.03
Black	112 (12.1)	370 (12.8)		460.2 (11.9)	471.1 (12.4)	
Other	13 (1.4)	56 (1.9)		59.3 (1.5)	66.9 (1.8)	
Pacific Islander	2 (0.2)	12 (0.4)		15.7 (0.4)	14.2 (0.4)	
White	761 (82.4)	2348 (81.2)		3184.4 (82.2)	3115.3 (81.9)	
Insurance status (%)			0.16			0.04
Medicare/Medicaid	66 (7.2)	247 (8.5)		353.9 (9.1)	316.3 (8.3)	
Military/VA	314 (34.0)	800 (27.7)		1079.7 (27.9)	1096.4 (28.8)	
No insurance	15 (1.6)	48 (1.7)		60.3 (1.6)	62.4 (1.6)	
Other	20 (2.2)	43 (1.5)		69.9 (1.8)	67.0 (1.8)	
Private	502 (54.4)	1723 (59.6)		2272.0 (58.6)	2228.7 (58.6)	
Unknown	6 (0.7)	29 (1.0)		39.2 (1.0)	35.2 (0.9)	
Median income (%)			0.07			0.06
>$74,063	341 (36.9)	1044 (36.1)		1314.5 (33.9)	1368.7 (36.0)	
$46,277–$57,856	146 (15.8)	450 (15.6)		594.7 (15.3)	584.9 (15.4)	
$57,857–$74,062	168 (18.2)	601 (20.8)		830.5 (21.4)	768.5 (20.2)	
<$46,277	118 (12.8)	370 (12.8)		513.4 (13.2)	488.7 (12.8)	
Unknown	150 (16.3)	425 (14.7)		621.9 (16.0)	595.0 (15.6)	
Histology (%)			0.10			0.02
IPC	52 (5.6)	160 (5.5)		192.4 (5.0)	201.2 (5.3)	
IDC	712 (77.1)	2331 (80.7)		3112.4 (80.3)	3039.8 (79.9)	
ILC	83 (9.0)	219 (7.6)		326.9 (8.4)	317.3 (8.3)	
MC	7 (0.8)	15 (0.5)		24.7 (0.6)	23.6 (0.6)	
Other	69 (7.5)	165 (5.7)		218.5 (5.6)	224.1 (5.9)	
HR status (%)			0.04			0.02
Negative	149 (16.1)	506 (17.5)		636.0 (16.4)	657.8 (17.3)	
Positive	774 (83.9)	2384 (82.5)		3238.9 (83.6)	3148.1 (82.7)	
Stage (%)			0.28			0.03
I	491 (53.2)	1143 (39.6)		1632.7 (42.1)	1626.0 (42.7)	
II	284 (30.8)	1120 (38.8)		1402.4 (36.2)	1398.5 (36.7)	
III	148 (16.0)	627 (21.7)		839.8 (21.7)	781.5 (20.5)	
Grade (%)			0.40			0.02
1	185 (20.0)	239 (8.3)		395.6 (10.2)	411.4 (10.8)	
2	425 (46.0)	1268 (43.9)		1742.1 (45.0)	1692.6 (44.5)	
3	313 (33.9)	1383 (47.9)		1737.2 (44.8)	1702.0 (44.7)	
Charlson–Deyo score (%)			0.07			0.06
0	803 (87.0)	2467 (85.4)		3289.8 (84.9)	3265.0 (85.8)	
1	93 (10.1)	343 (11.9)		499.7 (12.9)	438.5 (11.5)	
2	18 (2.0)	44 (1.5)		55.9 (1.4)	59.3 (1.6)	
3	9 (1.0)	36 (1.2)		29.5 (0.8)	43.1 (1.1)	
Surgery type (%)			0.18			0.03
Lumpectomy	632 (68.5)	1726 (59.7)		2324.9 (60.0)	2329.7 (61.2)	
Mastectomy	291 (31.5)	1164 (40.3)		1550.0 (40.0)	1476.2 (38.8)	
Adjuvant chemotherapy (%)			1.23			0.01
No	573 (62.1)	336 (11.6)		902.8 (23.3)	901.0 (23.7)	
Yes	350 (37.9)	2554 (88.4)		2972.1 (76.7)	2905.0 (76.3)	
Neoadjuvant therapy (%)			0.40			0.03
No	738 (80.0)	2693 (93.2)		3452.7 (89.1)	3424.9 (90.0)	
Yes	185 (20.0)	197 (6.8)		422.2 (10.9)	381.0 (10.0)	

IPC, Intraductal Papillary Carcinoma; IDC, Invasive Ductal Carcinoma; ILC, Invasive Lobular Carcinoma; MC, Mucinous Carcinoma; HR, Hormone Receptor.

## Data Availability

The dataset analyzed in this study was obtained from the National Cancer Database (NCDB). All analytical results generated during this study are presented in the manuscript.

## Supplementary Materials

The following supporting information can be downloaded at: https://www.mdpi.com/article/10.3390/cancers18071145/s1, Figure S1: Comparison of adjustment methods. Graphical comparison of covariate balance achieved using inverse probability of treatment weighting (IPTW) and propensity score matching (PSM); Figure S2: Covariate balance before and after IPTW adjustment. Standardized mean differences for baseline characteristics before and after inverse probability of treatment weighting, demonstrating improvement in covariate balance following adjustment; Figure S3: Forest plot of prespecified subgroup analyses; Figure S4: Subgroup analysis by metastatic sites in stage IV patients. Exploratory analysis of sequencing strategies stratified by specific metastatic sites among stage IV inoperable patients.

## Abbreviations

The following abbreviations are used in this manuscript:

CI	Confidence Interval
cGAS	Cyclic GMP-AMP Synthase
DC	Dendritic Cell
HR	Hazard Ratio
HER2	Human Epidermal Growth Factor Receptor 2
IDC	Invasive Ductal Carcinoma
ILC	Invasive Lobular Carcinoma
IPC	Intraductal Papillary Carcinoma
IPTW	Inverse Probability of Treatment Weighting
IR	Immunotherapy-first Sequencing
KM	Kaplan–Meier
MC	Mucinous Carcinoma
MHC	Major Histocompatibility Complex
NCDB	National Cancer Database
OS	Overall Survival
PD-L1	Programmed Death Ligand 1
PSM	Propensity Score Matching
RI	Radiotherapy-first Sequencing
SMD	Standardized Mean Difference
STING	Stimulator of Interferon Genes
