# Supplementary material for: Exploration of Optimal Synergistic Treatment Strategies of Postoperative Radiotherapy and Immunotherapy in Early-Stage Breast Cancer
Source: Cancers (Basel). 2026 Apr 2;18(7):1145. doi: 10.3390/cancers18071145 (PMC13072069; doi:10.3390/cancers18071145)
Supplement: Supplementary file 1 [file cancers-18-01145-s001.zip › cancers-4174923-supplementary.pdf]

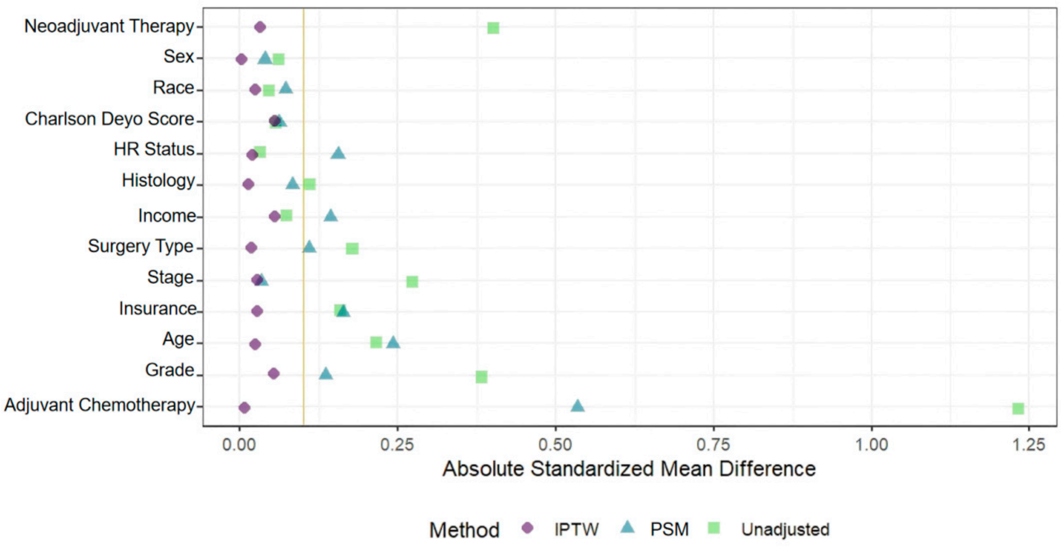

Figure S1. Comparison of adjustment methods. Graphical comparison of covariate balance achieved using inverse probability of treatment weighting (IPTW) and propensity score matching (PSM).

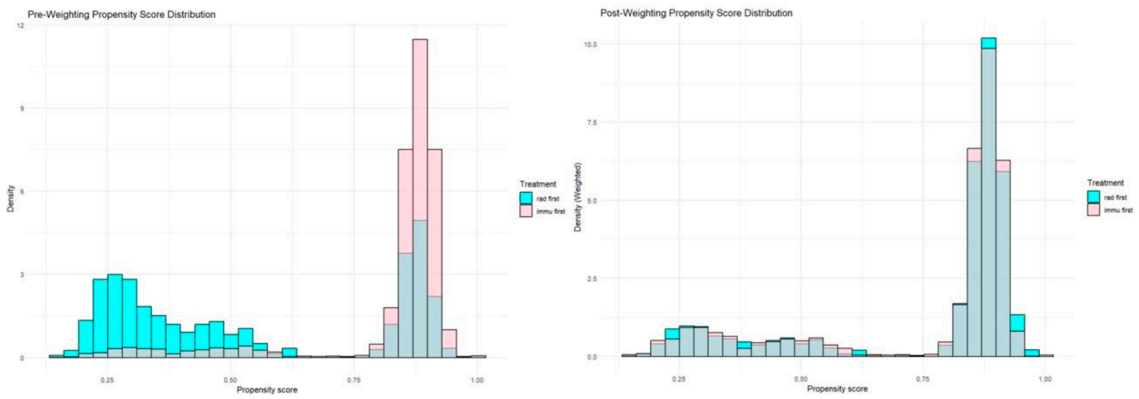

Figure S2. Covariate balance before and after IPTW adjustment. Standardized mean differences for baseline characteristics before and after inverse probability of treatment weighting, demonstrating improvement in covariate balance following adjustment.

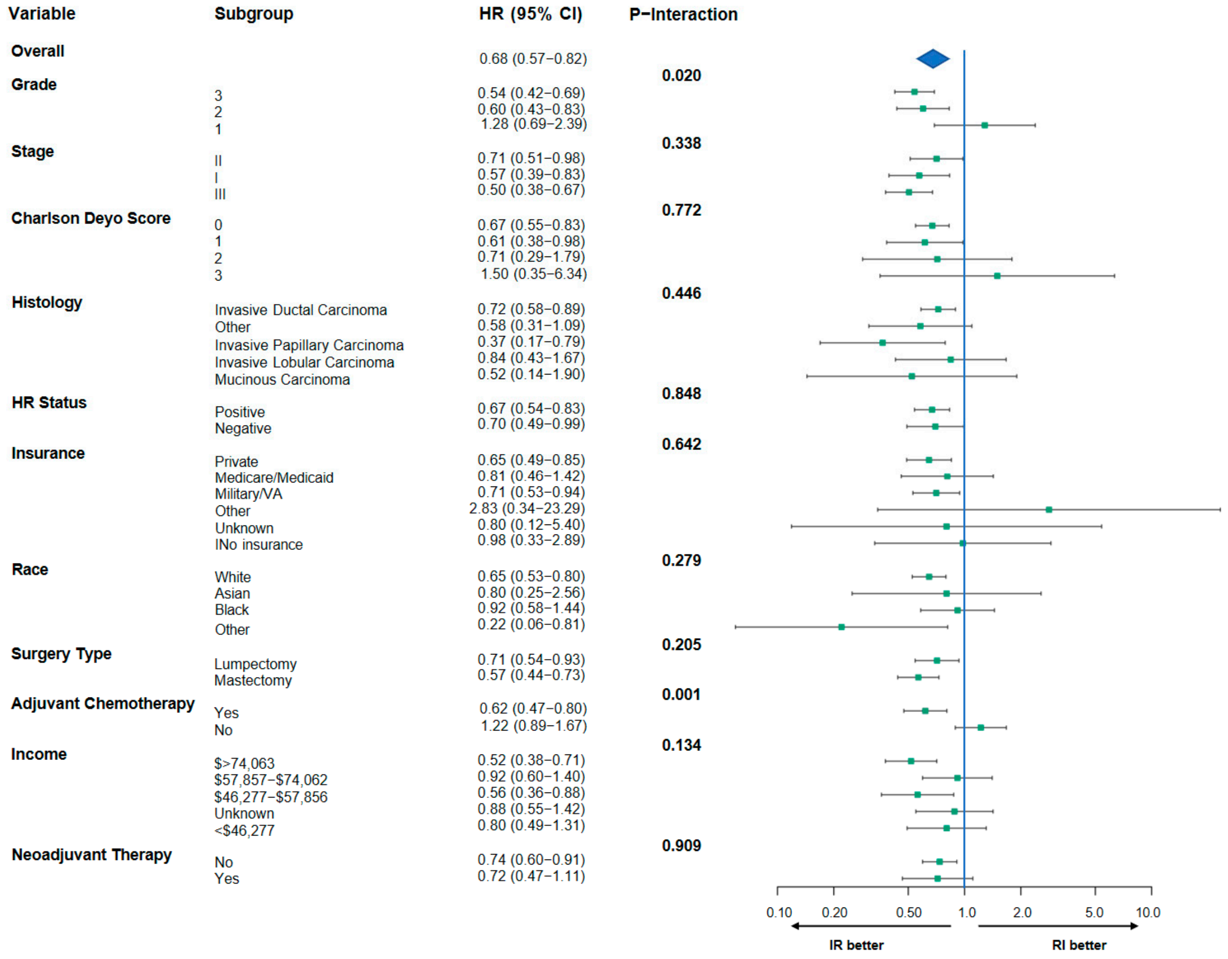

Figure S3. Forest plot of prespecified subgroup analyses.

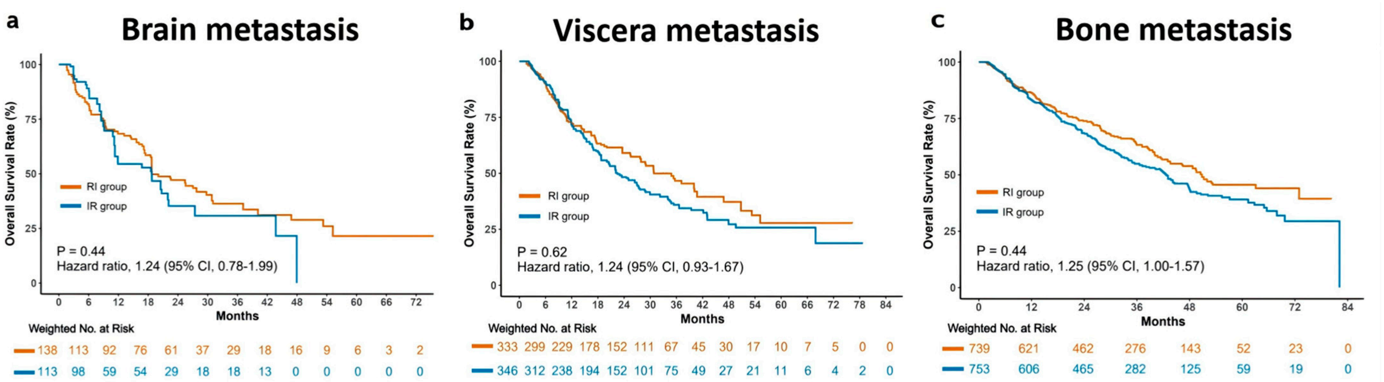

Figure S4. Subgroup analysis by metastatic sites in stage IV patients. Exploratory analysis of sequencing strategies stratified by specific metastatic sites among stage IV inoperable patients.
